# Persuasive Effects of Crisis Communication during Public Health Emergency Outbreaks in China

**DOI:** 10.3390/bs14100885

**Published:** 2024-10-01

**Authors:** Ting Wu, Guang Yu

**Affiliations:** School of Management, Harbin Institute of Technology, Harbin 150001, China; wuting1248@stu.hit.edu.cn

**Keywords:** crisis communication, persuasive effect, public health emergencies, emotion analysis, online media

## Abstract

Major global public health emergencies face unprecedented challenges, such as an infodemic and scientific disputes, and governments especially need to implement fast and effective crisis communication. Firstly, this paper takes the Elaboration Likelihood Model as a framework and constructs a crisis communication persuasion effect evaluation method with emotion analysis. Secondly, this paper takes the crisis communication at the beginning of the COVID-19 outbreak in China as an example and examines the persuasive effects of the peripheral route, represented by medical experts, and the central route, represented by mainstream media. This study finds that the peripheral route of persuasive communication can quickly establish communication trust and quickly change the public’s peripheral attitude, but the persuasive effect is unstable. The central route of persuasive communication demonstrates a significantly positive, stable, and anti-interference persuasive effect. Dual-subject persuasion is an important strategy for controlling an outbreak by rapidly establishing communication trust, combating an infodemic, boosting public confidence, and popularizing medical knowledge. This study evaluates the persuasive effects of crisis communication in the early stages of the COVID-19 outbreak in China, hoping to provide valuable practical references for crisis communication during the outbreak in future global public health emergencies.

## 1. Introduction

With the popularization of online media (e.g., social media, short videos, and live streaming) and AI technology, the convenience of obtaining information from online media has increased, which has also led to the widespread and rapid dissemination of health-related misinformation [1,2]. As a result, global public health emergencies face unprecedented crisis communication challenges. For example, during the COVID-19 outbreak, an infodemic [3] severely affected the implementation of outbreak prevention and control measures, such as Non-Pharmaceutical Interventions (NPIs) (e.g., mask protection and social distancing restrictions) [4,5], and the popularization of medical knowledge. An infodemic is triggered by the rapid spread of false information on social media [6,7,8,9]. An infodemic not only negatively affects widely adopted health protection behaviors such as NPIs [10,11,12,13] but also jeopardizes public behavioral patterns and health [13,14,15]. In the context of the interaction between public behavior and viral transmission, the infodemic became one of the key influencing factors in containing the spread of COVID-19 [16,17,18].

Scientific conflict was also one of the important factors that severely affected crisis communication in the early stages of the COVID-19 outbreak, where inconsistency between governments and medical experts was particularly problematic [19]. Scientific conflict, the downplaying of threats [20], the untimely release of information, and the lack of trust in agencies and the media affected public perceptions of risk, government trust, and compliance with NPIs [21,22]. For example, in the United States, President Trump violated the principles of public health (e.g., maintaining transparency and deferring to medical experts) [23] by dismissing important administrative expertise [24]. In China, local governments downplayed threats, failed to release information in a timely manner, lacked professional support, and incorrectly treated medical personnel such as Li Wenliang as rumor mongers, hindering the dissemination of scientific expertise [25]. The dissemination of misleading information, such as in the Shuanghuanglian incident, undermined the credibility of scientific expertise [12].

In addition, in the early stages of the COVID-19 outbreak, China faced the risk of spreading the virus as a result of massive population migration during the Spring Festival. The convergence of the triple factors of population migration, infodemic, and scientific conflict posed a very special challenge to the Chinese government’s crisis communication. There is an urgent need for the Chinese government to communicate information quickly, accurately, and effectively [26,27] to curb the development of an infodemic in a short period of time, to convince the public to act in unison and comply with health-protecting behaviors, and ultimately to contain the epidemic.

Regarding the threat of an infodemic, scientific conflicts, and other challenges in the context of major epidemic crisis communication, many scholars have explored related issues. With the rapid development of new online media, scholars are interested in the role these platforms play in crisis communication. According to Wang Y. et al., a variety of online media (e.g., Sina Weibo, TikTok, Twitter, Facebook, and Instagram) played an important role in crisis communication and helped inhibit the spread of the COVID-19 virus [19,28,29,30,31,32,33]. Online media can quickly convey real information [21] and help build trust between the government and the public [34]. For example, in China, the central government-affiliated mainstream media, CCTV, established a new model of 24-hour livestream dissemination of information on the construction of Leishenshan and Huoshenshan cabin hospitals; this form of news reporting is characterized by a wide range of influences and a high degree of public participation, which has effectively boosted the public’s confidence and enhanced the government’s credibility [25]. Various online media became important and effective platforms for crisis communication during the COVID-19 pandemic.

On the other hand, the government and medical experts were the main information sources for the public [35,36]. Among them, medical experts gained public trust through their posted content [37] and played a prominent persuasive role on social media such as Twitter [35,38]. In fact, compared with the government, the information posted by medical experts was more popular and recognized by the public (such as retweets and likes) [32]. In the early stages of the COVID-19 outbreak, governments had to adopt an expert-centered approach [23] to addressing scientific conflicts by leveraging the key role of medical experts in crisis communication. For instance, in Italy, specialized technical advice played a key policy role, and it almost completely overlapped with the political response [39]. In addition, the mainstream media remained an influential source of information that governments could use to disseminate correct news and combat false information [28,40]. The expertise of medical experts and the influence of government-affiliated mainstream media play a key role in crisis persuasive communication.

In the early stages of the COVID-19 outbreak in China, the crisis communication practices of medical experts and mainstream media attracted great attention. For example, medical experts such as Zhong Nanshan released messages clarifying facts, popularizing medical knowledge and NPIs, and attracting widespread public attention on social media. Additionally, the mainstream media, CCTV, established a 24-hour livestream to broadcast the construction progress of Leishenshan and Huoshenshan cabin hospitals, with an average of more than 100 million daily viewers [25]. As exemplified by Chinese practice, exploring the crisis communication effectiveness of dual-subject persuasion is an important reference value for future global public health emergencies.

Here, this paper presents a retrospective analysis of crisis communication on online media during the COVID-19 outbreak in China. Specifically, based on the Chinese social media platform, Sina Weibo, it examines the persuasive effects of the crisis communication strategies implemented by medical experts and central government-affiliated mainstream media in this major public health emergency. The trending topics related to crisis communication of medical experts and mainstream media on Sina Weibo’s real-time hot search list (HSL) are selected as the research sample. Moreover, a persuasive effect conceptual model of dual-subject persuasion is constructed based on persuasion theory. Then, this paper quantitatively evaluates the persuasive effects of the communication strategies implemented by medical experts and mainstream media. This paper hopes to provide references for crisis communication strategies for future global public health emergency outbreaks.

The rest of this paper is organized as follows: the second part describes the persuasion theory model and persuasive effect evaluation method; the third part presents the experiments designed for evaluating the persuasive effect of the communication strategies implemented by medical experts and mainstream media; the fourth part presents an analysis of the experimental results obtained; the fifth part is a discussion of the results; lastly, the conclusion of this paper is presented in the sixth part.

## 2. Theories and Methods

### 2.1. Theories

It has been shown that the persuasive effect is related to the way people process information, which is based on a dual-process model, and results in behavioral changes through attitude changes [41]. The Elaboration Likelihood Model (ELM) is a commonly used dual-process model that can appropriately describe the public’s information processing during a crisis [42]. According to the ELM, people’s information processing patterns are divided into the central and peripheral routes [43]. The central route emphasizes the role of people’s motivation and ability to process information and assumes that people will think systematically in an effortful and detailed way, thus forming or strengthening their central attitudes, regardless of whether they change or not. Attitudes formed by adopting the central route are thus enduring and resistant. In the peripheral route, people process information with very little detail. When people lack the motivation or ability to process information, they passively rely on peripheral cues (e.g., attractive or expert sources, media richness, and the number of arguments) and use cognitive shortcuts to form their peripheral attitudes, which are thus relatively temporary and susceptible.

This paper defines this dual process in crisis communication within the ELM framework. In the early stages of the COVID-19 outbreak, due to the lack of a priori knowledge, the public was motivated but lacked the ability to process information related to COVID-19. According to the ELM, the public first relies on peripheral cues (e.g., expert sources and online media) and selects cognitive shortcuts to process information. In this paper, the peripheral route is defined as the persuasive communication process that is dominated by medical experts. In the early stages of the COVID-19 outbreak, three medical experts, Zhong Nanshan, Li Lanjuan, and Zhang Wenhong, played important roles in fighting against the epidemic at the central and local government levels. Notably, Zhong Nanshan already had a high reputation among the public for his key role in the 2003 SARS outbreak, and when he took on the role of head of the central government’s High-Level Expert Group on Combating COVID-19 for crisis communication, he attracted remarkable attention from the public. Li Lanjuan and Zhang Wenhong are both senior experts in the field of infectious diseases, although they were not well known to the public before the COVID-19 outbreak. However, their crisis communication and actions during the COVID-19 outbreak generated a lot of attention in social media. This paper takes these three influential medical experts and uses their crisis communication topics on social media as peripheral cues to develop a study on the persuasive effect of the peripheral route.

**Figure 1:**
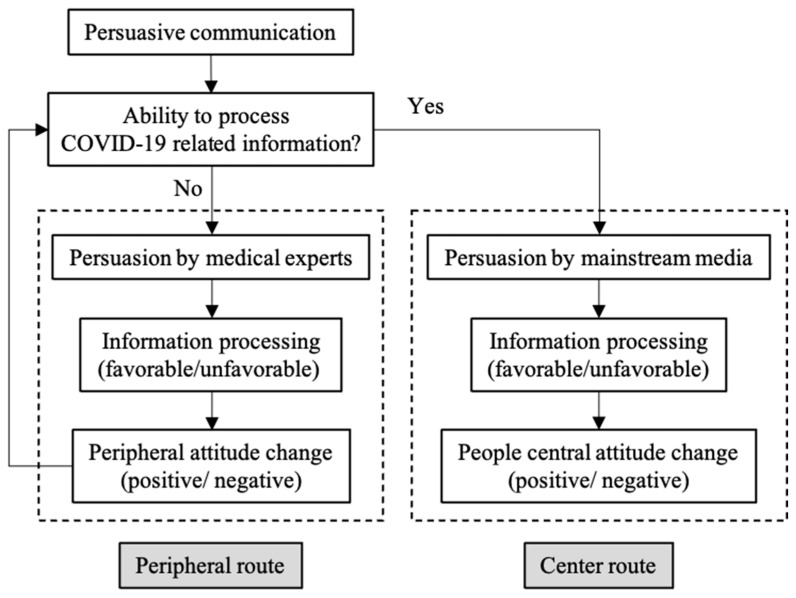
ELM-based dual-subject persuasion routes.

According to the ELM, peripheral attitudes are relatively temporary and susceptible. In order to ensure that the public can comply with the government’s anti-epidemic measures, such as NPIs, in an enduring and resistant manner, persuasive communication through the central route is needed after the peripheral route has taken effect. In the early stages of the COVID-19 outbreak, the central government-affiliated mainstream media implemented an innovative persuasive communication strategy by utilizing new media technology. This provided a platform for the public to independently process information related to government measures and freely express their opinions and attitudes. In this paper, the central route is defined as persuasive communication conducted by the central government-affiliated mainstream media by using the above-mentioned technology. For convenience, this article refers to the central route as mainstream media. In the early stages of the COVID-19 outbreak, the local government of Wuhan built two new cabin hospitals, Leishenshan Hospital and Huoshenshan Hospital, to treat COVID-19 patients, with more than 30,000 workers involved in the hospitals’ construction; the two buildings were finished in 10 days and 12 days, respectively, which was attributed to the commendable speed of the Chinese government. At the same time, the central government-affiliated mainstream media, CCTV, established a 24-hour livestream, also named “slow live broadcast”, to air the construction progress in real time by fixing two fixed cameras at each hospital construction site (without voice-over or narration, but only construction site sound). Mainstream media limited the topic to these cabin hospitals’ construction, and the public could freely express their views and organize sub-topics by commenting on the pop-ups. The average daily number of viewers of this slow live broadcast exceeded 100 million and caused extensive discussion on Sina Weibo, making it the most popular anti-epidemic event on social media [44], which is a typical case of crisis communication. In the context of the need for fast, accurate, and effective persuasive communication, this paper takes the slow live broadcast established by CCTV as the object of study and investigates the persuasive effect of the central route by analyzing the sub-topics and attitudes expressed by the public. The ELM-based dual-subject persuasion routes are constructed, as shown in Figure 1.

### 2.2. Persuasive Effect Evaluation Method Based on Pre-Training Model

The implementation of the ELM involves the use of public attitude change to evaluate the persuasive communication effect [43]. According to Liu S.R. et al., emotion analysis is an important tool for studying public attitude [45], so this paper uses the public’s emotion distribution in comments and retweets collected to measure their attitude. The methods of emotion analysis have evolved along with the development of machine learning, deep learning, and pre-training models [46,47,48]. It is noteworthy that pre-training models based on the Transformer structure have achieved better results than those based on machine learning and deep learning in various natural language processing tasks, including emotion analysis. Therefore, in this paper, the emotion analysis method is based on a pre-training model.

Transformer, the basic unit of pre-training models, is based on the self-attention mechanism [49], which can efficiently capture long-distance semantically dependent feature information, and is one of the preferred structures in the current natural language processing technology. The Bidirectional Encoder Representations from Transformers (BERT), proposed by Google in 2018 [50], was the first pre-training model that used the Transformer structure and achieved the best results in various Natural Language Processing (NLP) tasks that year. In the following two years, many improved models appeared under the framework of BERT, such as A Robustly Optimized BERT Pretraining Approach (RoBERTa) [51].

**Figure 2:**
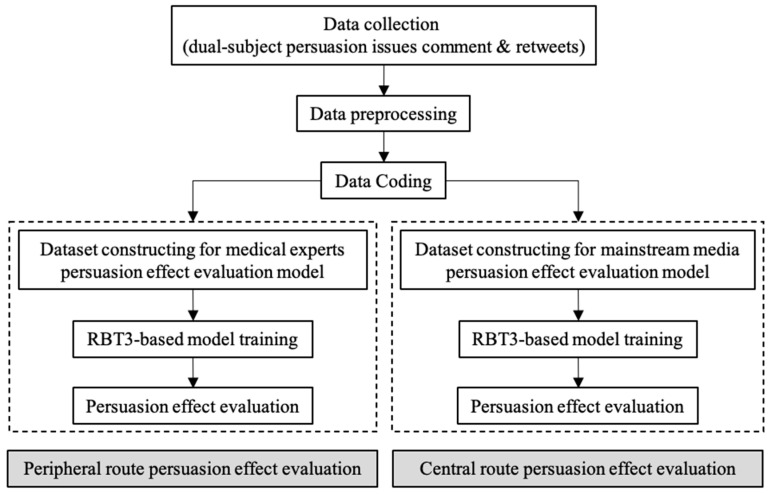
Process of evaluation of two-subject persuasive effects based on RBT3.

On the other hand, BERT-Chinese, released by Google, uses words as a granularity for word segmentation, which destroys the semantic connection between Chinese characters. To better adapt to the Chinese context, the Joint Laboratory of HIT and iFLYTEK Research (HFL) proposed a whole word masking Chinese pre-training model, RoBERTa-wwm-ext, based on RoBERTa pre-training model of Facebook [52]. It uses HFL’s Chinese language technology platform (LTP) as a tool for word segmentation, with significantly better performance than BERT. At the same time, in order to reduce the threshold for application, HFL streamlines model parameters and releases a small model, RBT3, based on the 3-layer Transformer. This model’s parameters are only 37.3% of RoBERTa-wwm-ext, and it can still achieve an effect comparable to Google’s BERT-base. In this study, RBT3 is ultimately selected as the method for emotion analysis by considering the model performance as well as the computational cost. The process of evaluating two-subject persuasive effects based on RBT3 is shown in Figure 2.

## 3. Empirical Study

### 3.1. Data Collecting

**Figure 3:**
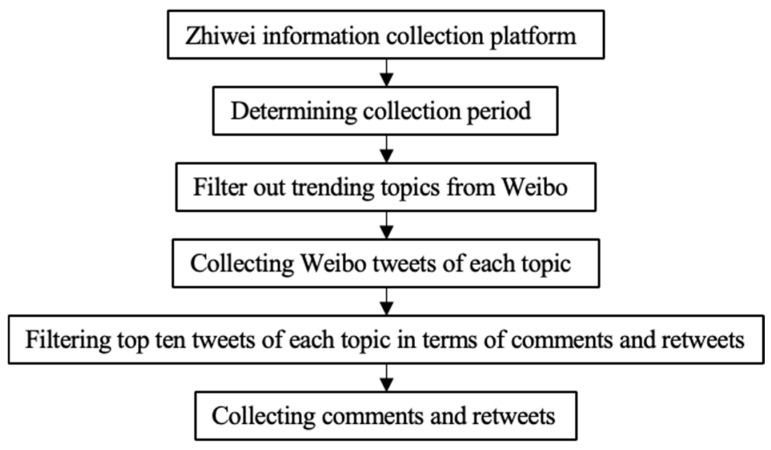
Data collection process.

This paper selects three representative medical experts in China as the research subjects, namely, Zhong Nanshan and Li Lanjuan, the leader and member, respectively, of the High-Level Expert Group of the National Health Commission, and Zhang Wenhong, the leader of the Shanghai COVID-19 Medical Treatment Expert Group. Secondly, this paper selects the central government-affiliated mainstream media, CCTV, and takes the slow live broadcast “Leishenshan and Huoshenshan Hospitals” as the research object. The data collection process is shown in Figure 3.

This study uses the hot search machine and information collection platform of Zhiwei Research Institute for data collection [53]. The Zhiwei information collection platform is a platform specially designed for university research and can collect all public information published on the Internet, except for data with privacy settings. This platform provides keyword-based data collection functions on multiple Internet platforms, including Sina Weibo. As long as researchers provide collection keywords, platform names, collection cycle, and other information, the platform outputs result in an Excel file format. Moreover, the collected data covers multiple dimensions, such as release time, title, content, publisher, and release platform. As part of this platform’s functions, comment and retweet collection can also be performed by providing, for example, the network link of a Sina Weibo tweet to obtain all the related comments and retweets.

Firstly, the data collection period was determined. By the end of February 2020, COVID-19 had been put under control in most provinces, and the critical period of crisis communication was nearly over. Therefore, the data collection period is from 1 January 2020 to 29 February 2020. Then, we determined the collection keywords. We utilized the hot search machine of Zhiwei Research Institute to filter out trending topics related to medical experts and mainstream media from Sina Weibo’s real-time hot search list. We found that the search volume of trending topics shows a discontinuous distribution, with the first tier having a much higher volume than the second tier. For medical experts, the first tier, containing four or five topics, yielded over three million results, and for the slow live broadcast, the first tier, containing seven topics, exceeded tens of millions of results. We only selected the trending topics of the first tier, as they have gained wider public attention. The selected topics are shown in Table 1, and the types of topics are shown in Table 2.

By using the above topics as keywords, the Weibo tweets on each topic were collected with the Zhiwei information collection platform. According to statistical analysis, for each topic, the top ten Sina Weibo tweets in terms of comments and retweets accounted for about 80% of the total, so these were selected for collection, resulting in a total of 304,246 comments and retweets.

### 3.2. Data Preprocessing

The collected Sina Weibo comments and retweets were preprocessed. First of all, the collected comments and retweets were unstructured data, containing research-irrelevant dimensions such as links, from which research-related data dimensions needed to be extracted. We used the Python programming language, and, based on the pandas library, the release time, content, and the number of likes were extracted. Secondly, the Python programming language was used to clean data, discarding abnormal data such as missing values, duplicate values, special characters, and topic-irrelevant noise data. In this research, we used expert judgment to determine the type and characteristics of the subject-independent noise data, built a dictionary, constructed an automated de-noising process, deleted subject-independent noise data using Python programming, and then used pandas and regular expressions to remove abnormal data. After cleaning, 301,819 valid comments and retweets for the 20 topics in Table 1 were obtained.

### 3.3. Experimental Process of Persuasive Effect Evaluation

This section begins with coding the emotion categories of the public comments. Although there is still no consensus among academics on emotion classification, many researchers agree on the six major emotion categories (anger, fear, sadness, happiness, disgust, and surprise) based on Affective Events Theory (AET) [54]. Therefore, this paper applies AET for data coding. (1) Filtering of emotion categories. We found that there is an imbalance in the data of different emotion categories, so those that do not have enough occurrences were excluded. Specifically, for medical experts, “happiness”, “disgust”, and “surprise” were excluded, and for mainstream media, “disgust” and “fear” were excluded. (2) Adjusting emotion categories. A large proportion of the comments and retweets showed the public’s support for the medical experts’ persuasion topics. Therefore, the category of “support“ was added to the coding. In addition, the corpus of the medical experts and mainstream media contain a large number of comments and retweets that are only statements of facts, devoid of any influence of emotions or stances, so the category of “objectivity“ was added to the coding. Then, we randomly sampled and coded the public comments and retweets related to the persuasive topics of medical experts and mainstream media. The emotion coding and the number of coding for medical experts and mainstream media are shown in Table 3 and Table 4, respectively.

Secondly, we utilized the above coding data to construct a dataset for training the evaluation model. We adopted a sampling method to solve the problem of data imbalance among those emotion categories. We carried out random sampling for emotion categories with a large number of data and oversampling for those with a small number of data, and the sampled data ultimately formed the dataset for training the evaluation model. Finally, we used random sampling to divide this dataset into a training set and a testing set and conducted model training with RBT3.

After sampling, for medical experts, the number of data points for each emotion category is 3000, and the constructed dataset has a total of 15,000 data points. After random sampling, the training set has 12,500 entries and the test set has 2500. After training, the accuracy (Acc) is 0.702, the recall is 0.702, F1 is 0.705, and the precision (P) is 0.732. The RBT3-based evaluation model for medical experts was denoted as Model 1. For mainstream media, the number of data points for each emotion category after sampling is 2500, and the constructed dataset has a total of 12,500 data points. After random sampling, the training set has 11,250 entries and the test set has 1250. After training, the accuracy (Acc) is 0.835, the recall is 0.835, F1 is 0.832, and the precision (P) is 0.849. The RBT3-based evaluation model for mainstream media was denoted as Model 2. Finally, we utilized Model 1 and Model 2 to categorize the uncoded comments and retweets relative to the topics of medical experts and mainstream media, respectively.

## 4. Results

This section presents the results of the emotion distribution and thematic analyses of the persuasive effects of peripheral and central routes, represented by medical experts and mainstream media, respectively.

### 4.1. Analysis of Persuasive Effect of Peripheral Route

#### 4.1.1. Emotion Distribution in Public Responses to the Peripheral Route

**Figure 4:**
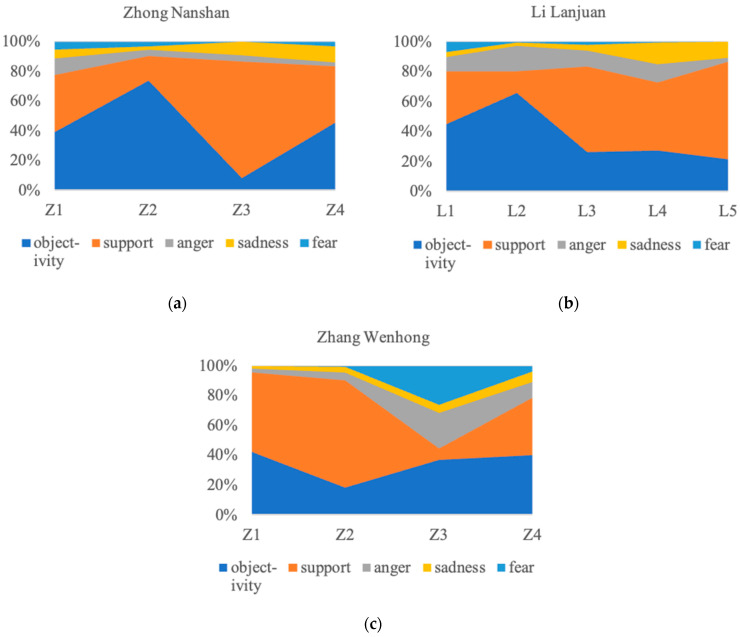
Emotion distribution in public responses to medical experts. (**a**–**c**) respectively represent the emotion distribution in public responses to Zhong Nanshan, Li Lanjuan, and Zhang Wenhong.

The emotion distribution in the public comments and retweets in response to the medical experts is shown in Figure 4. Overall, for the peripheral route, support and objectivity constitute the main body of public emotions. For Zhong Nanshan and Li Lanjuan, the sum of the percentages of these two categories is stable, with both being close to or over 80%. On the other hand, for Zhang Wenhong, the emotion distribution is polarized, with the sum of support and objectivity varying between about 45% and 90%, while in individual topics, anger and fear jointly constitute the dominant emotions.

According to the analysis by topic type, emotions of support are predominant in “frontline anti-epidemic”, indicating that the selfless and courageous behavior of medical experts has gained public recognition. This suggests that through this topic, Zhong Nanshan strengthens his reputation, while Li Lanjuan and Zhang Wenhong build up their reputations. Moreover, they all establish communication trust with the public. For topics classified as “clarifying facts”, emotions of objectivity dominate, followed by those of support and anger. For topics classified as “medical advice”, such as NPIs, objectivity and support jointly constitute the dominant emotions.

For Zhong Nanshan, Topic 1 and Topic 4 present close percentages of support and objectivity. Topic 3 is dominated by support (nearly 80%), while Topic 2 is dominated by objectivity (over 70%). For Li Lanjuan, Topics 3, 4, and 5 are all dominated by support (all over 45%), while Topic 1 and Topic 2 are dominated by objectivity (all over 45%). The exception is that Topic 2 is the only topic where anger exceeds support, with a percentage of 17%. For Zhang Wenhong, Topic 1 and Topic 2 are both dominated by support (all over 50%), and Topic 4 is dominated by objectivity and support (nearly 40% each). The difference is that the emotions of Topic 3 are mainly distributed among objectivity, anger, and fear (all over 24%), while anger and fear account for a combined total of 50%. Thus, there are abnormal emotion distributions with respect to the topics of medical experts.

#### 4.1.2. Thematic Analysis of Abnormal Emotion Distribution in Responses to the Peripheral Route

**Figure 5:**
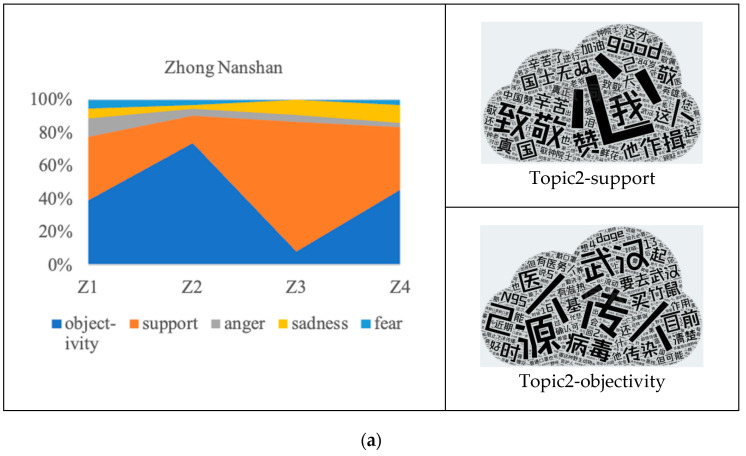

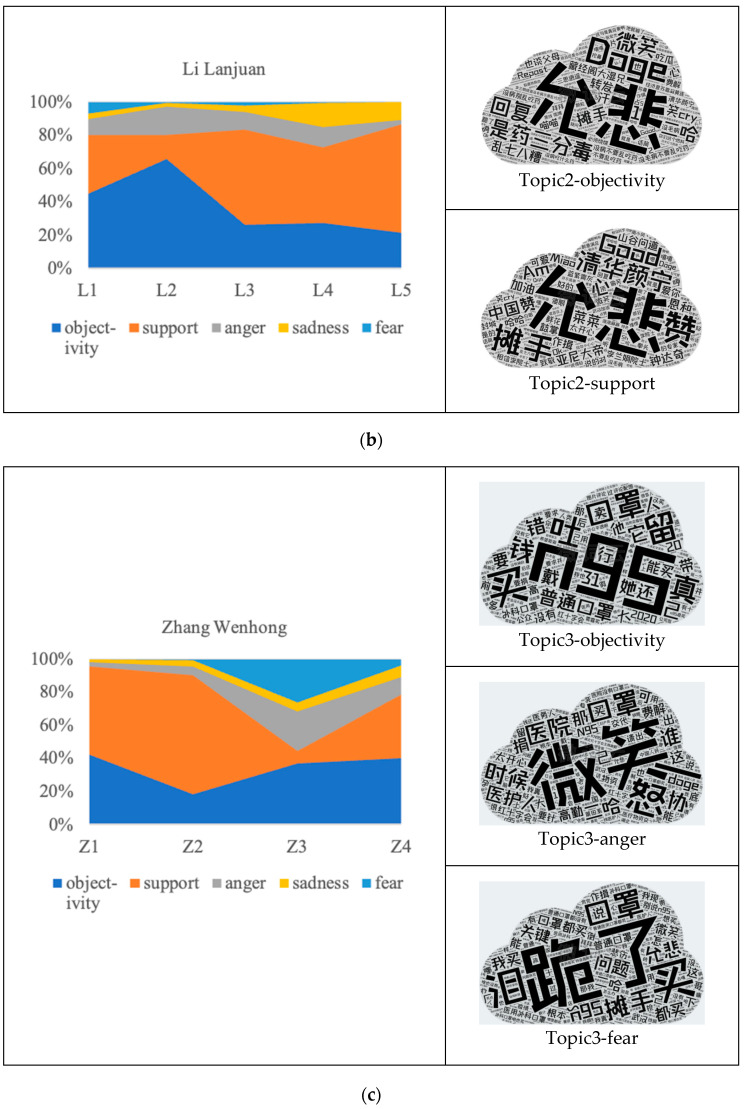
Thematic analysis of abnormal emotion distribution in public responses to medical experts. (**a**–**c**) respectively represent the thematic analysis results of abnormal emotion distribu-tion in public responses to Zhong Nanshan, Li Lanjuan, and Zhang Wenhong. The size of each word is directly proportional to its frequency of occurrence. Descending by frequency, the English ex-planations for the top five most frequently occurring words in each word cloud are provided. In (**a**), Topic2-objectivity: human-to-human transmission, source, Wuhan, oneself, and virus; Topic2-support: compassion, pay tribute, I, praise, and bow. In (**b**), Topic2-objectivity: please allow me to express my sorrow with a smile, doge meme, perfunctory smile, reply, and even medicine has its poison; Topic2-support: please allow me to express my sorrow with a smile, approval, shrug, Yan Ning from Tsinghua University, and good. In (**c**), Topic3-objectivity: N95 respirator, buying, retain, mask, and disgust; Topic3-anger: perfunctory smile, anger, hospital, mask, and medical staff; Topic3-fear: overwhelmed, buying, tears, mask, and shrug.

In order to further explain the abnormal emotion distributions, this paper conducts a thematic analysis of the public’s comments and retweets, and the results of the thematic analysis are shown in Figure 5.

For Zhong Nanshan, the emotion distributions in Topic 2 and Topic 3 fluctuate significantly. The proportion of objectivity in Topic 2 is as high as 73%, the highest among all topics. According to the word frequency analysis, the expression of objectivity mainly focuses on repeating Zhong Nanshan’s factual clarifications, as well as the changes in personal behavior (e.g., travel restrictions and dietary taboos). People tend to rationally focus on the facts of the epidemic itself and behavioral compliance such as NPIs. The proportion of support in Topic 3 is as high as 78%, the highest among all topics. According to the word frequency analysis, the expression of support mainly focuses on the support evaluation of Zhong Nanshan’s action (e.g., support and reverence), as a consequence of which this expert’s personal reputation is strengthened.

For Li Lanjuan, the emotion distributions in Topic 2 fluctuate significantly. The proportion of objectivity is nearly 66%, the highest among all topics, while the proportion of anger reaches 17%, which is close to the proportion of support. According to the word frequency analysis, the expression of objectivity focuses on repeating Li Lanjuan’s factual clarifications, while the expression of support expresses the public’s support for Li Lanjuan’s action. Additionally, the expression of anger shows a change in topic, whereby the public turns to express anger at the Wuhan Virus Institute and other related organizations for misleading the public.

For Zhang Wenhong, the emotion distributions are polarized. Support in Topic 1 and Topic 2 is absolutely the dominant emotion. In Topic 3, i.e., suggestions for epidemic prevention initiatives, the emotion distributions are reversed, with objectivity, anger, and fear being the dominant emotions. Through word frequency analysis, the expression of these three types focuses on repeating the factual clarification of mask shortage, attacking the Wuhan Red Cross, and manifesting the fear triggered by the shortage of supplies, respectively. Similar to Topic 2 for Li Lanjuan, changes in topics have also occurred, but they are not directed at Zhang Wenhong himself.

### 4.2. Analysis of the Persuasive Effect of Central Route

#### 4.2.1. Emotion Distribution in Public Responses to the Central Route

**Figure 6:**
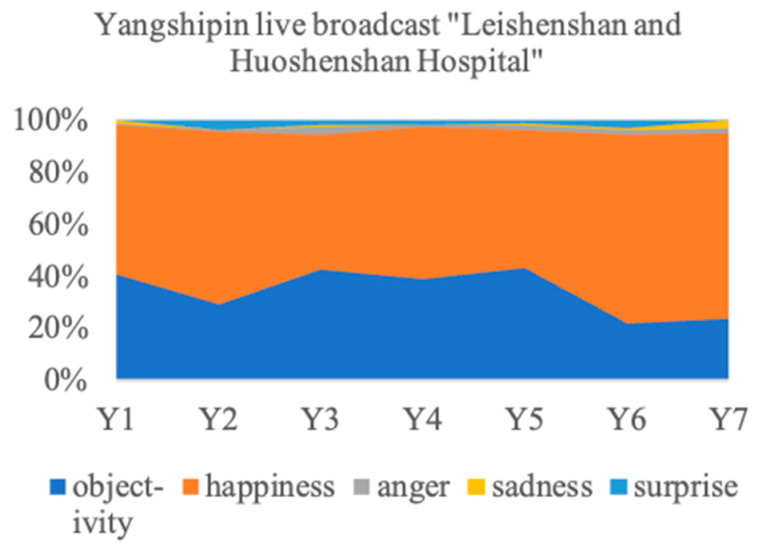
Emotion distribution in public responses to mainstream media.

The emotion distribution in the public comments and retweets in response to the communication strategies implemented by mainstream media is shown in Figure 6.

Unlike medical experts, there is an overall consistency in the public’s emotions associated with the slow live broadcast of mainstream media, as well as the negative topic (Topic 7). Overall, happiness and objectivity account for a combined total of more than 90%, constituting the bulk of public emotions. Among them, happiness accounts for more than 50% in all topics, occupying a dominant position. Although there are small fluctuations in the proportion of objectivity, it drops to its lowest value in the last two topics. Surprisingly, regarding the negative topic, happiness accounts for more than 70%, which is the second highest among all topics. This paper explores the reasons for the public’s anomalous attitude towards Topic 7 with a thematic analysis.

#### 4.2.2. Thematic Analysis of Abnormal Emotion Distribution in Public Responses to the Central Route

**Figure 7:**
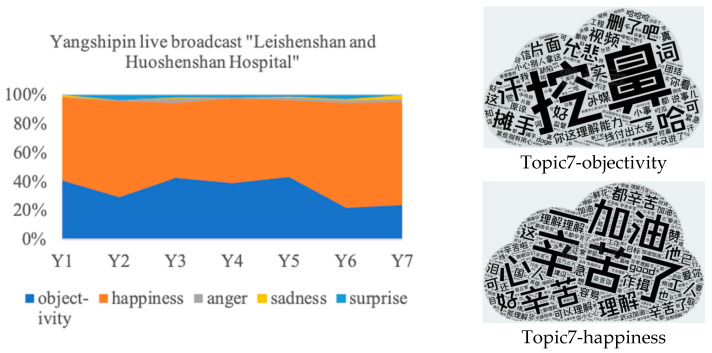
Thematic analysis of abnormal emotion distributions in public responses to mainstream media. The size of each word is directly proportional to its frequency of occurrence. Descending by fre-quency, the English explanations for the top five most frequently occurring words in each word cloud are provided. Topic7-objectivity: indifference, awkward, puzzled, shrug, and term; Top-ic7-happiness: hard work acknowledged, keep going, compassion, understand, and good.

The thematic analysis of Topic 7 is shown in Figure 7. The expression of happiness mainly focuses on the understanding and support for construction workers. Although this issue is caused by construction workers, it originated from their desire to speed up the construction process in the fight against COVID-19, i.e., it is a workplace error for the public interest. Conversely, the change in topics related to medical experts consists of denouncing behavior that disregards the public interest for the sake of personal or institutional self-interest. On the other hand, unlike the change in topics related to medical experts, the public did not express any recourse or dissatisfaction with the construction entity in Topic 7 and showed their tolerance, not meanness. This phenomenon can be ascribed to two primary factors. Firstly, notwithstanding the error committed, this action is perceived as being in alignment with the public interest. Secondly, after being exposed to the preceding six topics, the public’s psychological resilience to negative topics has been bolstered, facilitating a more rational and tolerant approach to processing negative information.

## 5. Discussion

According to the above findings, the peripheral central routes of crisis communication, represented by medical experts and mainstream media, respectively, show different persuasive effects.

### 5.1. Persuasive Effect of Peripheral Route Represented by Medical Experts

First of all, Zhong Nanshan, as the head of the high-level expert group of the National Health Commission, implemented crisis communication strategies at the macro-level, and his inherent reputation and personal charisma (participating in the fight against the epidemic at an old age) helped him establish communication trust with the public. The analysis results show that Zhong Nanshan’s persuasive effect is characterized by stability. Secondly, Li Lanjuan, as a member of the high-level expert group of the National Health Commission, implemented crisis communication strategies at the meso-level, and her personal charisma likewise helped her to build communication trust. As a whole, emotions of anger run through almost all topics in proportions of 10%, suggesting that her persuasive effect is at risk of being jeopardized by the potential evolution of topics. Thirdly, Zhang Wenhong, as the head of Shanghai’s COVID-19 medical treatment expert group, implemented crisis communication strategies at the micro-level, i.e., closer to the behavior and requirements of individuals. He quickly established communication trust through two frontline anti-epidemic topics. However, subsequent to this, there was a substantial fluctuation in the emotion distributions, increasing the risk posed by the possible evolution. In conclusion, these three medical experts succeeded in building communication trust based on their personal charisma and actions, and crisis communication topics on medical advice became progressively more personal and operational, gaining public support. However, the risk of the possible evolution of topics also increased, which may have weakened the persuasive effects of medical experts. Overall, the peripheral route of persuasive communication can quickly change the public’s peripheral attitudes, but the effect is temporary and susceptible. Thus, when adopting the peripheral route for persuasive communication, it is necessary to guard against the risk of potential issue evolution.

### 5.2. Persuasive Effect of Central Route Represented by Mainstream Media

The construction of the cabin hospitals was a key anti-epidemic measure, which played a decisive role in the prevention and control of COVID-19 in Wuhan and China. Mainstream media adopted the novel media model of slow live broadcast to show the progress of this critical measure in real time, with the aim of stabilizing and positively inspiring the public’s emotions. The analysis results show that broadcasting this progress encouraged significant participation among the public in a short period of time and demonstrated a significant positive persuasive effect. In the face of negative events, the construction entity intervened in social media in a timely manner and clarified the facts frankly. Compared with medical experts, the public tended to deal with negative information in a more focused, rational, and tolerant way under the persuasive communication strategies implemented by mainstream media, and there was no risk related to possible issue evolution. It shows that the central route has withstood the impact of negative events on crisis communication and demonstrates enduring and resistant persuasive effects.

## 6. Conclusions

This study demonstrates that the ELM-based dual-subject persuasion model consisting of medical experts and mainstream media ensured effective crisis communication in the early stages of the COVID-19 outbreak. By utilizing new media communication modes such as social media and slow live streaming, the two subjects leveraged their professionalism and influence, rapidly established communication trust, combatted the infodemic, and popularized medical knowledge in the early stages of the COVID-19 outbreak. This study reviews the cases of online media-based crisis communication in the early stages of the COVID-19 outbreak in China, and the findings have several important implications for future crisis communication practice.

Policymakers should first rely on medical experts for crisis communication, leveraging their expertise to quickly establish communication trust with the public and promptly disseminating medical information such as NPIs. At the same time, monitoring public attitudes is necessary to adjust expert communication strategies and prevent risks caused by changes in public attitudes. Secondly, mainstream media should utilize various influential new media means, such as slow live streaming and short videos, to timely showcase the government’s key anti-epidemic measures. In the next stage of crisis communication, it is necessary to dynamically adjust government measures and communication strategies based on the needs and attitudes expressed by the public.

This study has some limitations, and there is room for improvement with future in-depth research. Firstly, this study only explores the situation in the early stages of the outbreak, so the influence and role of medical experts and mainstream media in the later stages of the COVID-19 pandemic could be studied in the future. Secondly, the role and impact of the communication strategies implemented by medical experts and mainstream media are only analyzed in the Chinese context; in future research, they could be analyzed in the context of different countries and regions [55] to explore more effective mechanisms. Thirdly, although this study analyzes successful crisis communication strategies of medical experts and mainstream media, it will be necessary to explore the heterogeneity presented by these two subjects. Specifically, regarding the persuasive effects of the peripheral route, more types of experts and peripheral cues need to be taken into consideration. Regarding the persuasive effects of the central route, it will be essential to consider not only the persuasive communication cases from mainstream media but also influential persuasive communication cases from other online new media, such as short videos and live streaming. Finally, the data in this study are derived from social media only, so future research could explore cross-platform persuasive communication mechanisms.

## Figures and Tables

**Table 1:** Dual-subject persuasion topics on Sina Weibo.

	Zhong Nanshan	Li Lanjuan	Zhang Wenhong	Slow Live Broadcast “Leishenshan and Huoshenshan Hospitals”
Topic 1	Zhong Nanshan talks about the epidemic	Li Lanjuan responds to six questions about the COVID-19	Huashan Hospital	Network contractor
Topic 2	Zhong Nanshan affirms human-to-human transmission of the COVID-19	Li Lanjuan advocates not taking medicine indiscriminately if there is no problem	Shanghai medical expert team leader responds to party members on the front line	Watching the live broadcast of the hospital if you are bored and cannot sleep
Topic 3	84-year-old Zhong Nanshan fights again on the front line of epidemic prevention	Li Lanjuan responds to vaccine progress	Experts call for leaving N95 masks to medical staff	Cloud supervisor
Topic 4	Zhong Nanshan emphasizes that travel should be avoided at present	Li Lanjuan only sleeps three hours every day	Please resist for two weeks to smother the virus	Fork sauce and shovel sauce
Topic 5	——	The indentation on Li Lanjuan’s face	——	Excavator Sky Mission
Topic 6	——	——	——	Excavator help list
Topic 7	——	——	——	Huoshenshan conflict

**Table 2:** Types of dual-subject persuasion topics on Sina Weibo.

	Persuasion Subjects	Zhong Nanshan	Li Lanjuan	Zhang Wenhong	Slow Live Broadcast “Leishenshan and Huoshenshan Hospitals”
Types of Topics					
Frontline anti-epidemic		Topic 3	Topic 4–5	Topics 1–2	——
Clarifying facts		Topics 1–2	Topics 1–2	Topic 3	——
Medical advice		Topic 4	Topic 3	Topic 4	——
Public-generated sub-topics		——	——	——	Topics 1–6
Negative events		——	——	——	Topic 7

**Table 3:** Description of emotion coding for medical experts.

Emotion Coding	0	1	2	3	4
Emotion categories	objectivity	support	anger	sadness	fear
Number	2833	3589	2891	679	375

**Table 4:** Description of emotion coding for mainstream media.

Emotion Coding	0	1	2	3	4
Emotion categories	objectivity	happiness	anger	sadness	surprise
Number	1612	23,485	221	224	246

## Data Availability

The datasets generated during and/or analyzed during the current study are available in the Harvard Dataverse repository at https://doi.org/10.7910/DVN/I095TH.
